# How do the outcomes of the DEKA Arm compare to conventional prostheses?

**DOI:** 10.1371/journal.pone.0191326

**Published:** 2018-01-17

**Authors:** Linda J. Resnik, Matthew L. Borgia, Frantzy Acluche, Jill M. Cancio, Gail Latlief, Nicole Sasson

**Affiliations:** 1 Research Department, Providence VA Medical Center, Providence, Rhode Island, United States of America; 2 Health Services, Policy and Practice, Brown University, Providence, Rhode Island, United States of America; 3 Center for the Intrepid, Brooke Army Medical Center JBSA, Ft. Sam Houston, TX, United States of America; 4 Extremtiy Trauma and Amputation Center of Excellence, Ft. Sam Houston, TX, United States of America; 5 Department of PM&R, James A Haley Veteran’s Hospital, Tampa, FL, United States of America; 6 NYU School of Medicine, New York, New York, United States of America; 7 VA NY Health Harbor System, New York, New York, United States of America; Northwestern University, UNITED STATES

## Abstract

**Objectives:**

Objectives were to 1) compare self-reported function, dexterity, activity performance, quality of life and community integration of the DEKA Arm to conventional prostheses; and 2) examine differences in outcomes by conventional prosthesis type, terminal device type and by DEKA Arm configuration level.

**Methods:**

This was a two-part study; Part A consisted of in-laboratory training. Part B consisted of home use. Study participants were 23 prosthesis users (mean age = 45 ± 16; 87% male) who completed Part A, and 15 (mean age = 45 ± 18; 87% male) who completed Parts A and B. Outcomes including self-report and performance measures, were collected at Baseline using participants’ personal prostheses and at the End of Parts A and B. Scores were compared using paired t-tests. Wilcoxon signed-rank tests were used to compare outcomes for the full sample, and for the sample stratified by device and terminal device type. Analysis of outcomes by configuration level was performed graphically.

**Results:**

At the End of Part A activity performance using the DEKA Arm and conventional prosthesis was equivalent, but slower with the DEKA Arm. After Part B, performance using the DEKA Arm surpassed conventional prosthesis scores, and speed of activity completion was equivalent. Participants reported using the DEKA Arm to perform more activities, had less perceived disability, and less difficulty in activities at the End of A and B as compared to Baseline. No differences were observed in dexterity, prosthetic skill, spontaneity, pain, community integration or quality of life. Comparisons stratified by device type revealed similar patterns. Graphic comparisons revealed variations by configuration level.

**Conclusion:**

Participants using the DEKA Arm had less perceived disability and more engagement of the prosthesis in everyday tasks, although activity performance was slower. After home use experience, activity performance was improved and activity speed equivalent to using conventional prostheses.

## Introduction

As the availability of and consumer demand for more advanced and expensive upper limb prosthetic devices increases, so does the need for studies that compare the effectiveness of these devices. The DEKA Arm is an example of a new technologically advanced upper limb prosthesis. It was developed under the Revolutionizing Prosthetics Program through DARPA funding,[1] and approved by U.S. Food and Drug Administration in 2014. It has recently become commercially available and is being called the Luke Arm. [2]

The device is available in 3 configurations or levels: radial configuration (RC) for persons with radial amputation; humeral configuration (HC) for persons with humeral amputation; and shoulder configuration (SC) for persons with shoulder disarticulation, forequarter amputation or very short transhumeral amputation. [1] Unique features of all configuration levels are a powered wrist which allows flexion and extension combined with radial and ulnar deviation, powered wrist pronation and supination and six programmable hand grip patterns. The HC and SC have powered elbow flexion and extension and powered humeral rotation. The SC has additional powered shoulder movements and is controlled by Endpoint control which allows simultaneous joint control. [3] The DEKA Arm is controlled primarily with inertial measurement units (IMUs) secured to top of the shoes. However this control method may be supplemented by electromyography (EMG) controls, pressure transducers, and conventional controls such as linear transducers, and rocker or other switches. [4] All control options are configurable using a wireless Prosthetist interface that allows customization of the prosthetic actions/functions assigned to each control as well as the thresholds and gains of each control. Selection of control type, and configuration of those controls are guided by user preference and prosthetist judgement. In all cases, subjects used some type of switch or transducer (pressure transducer, linear transducer, rocker switch for mode selection (HC level) and for going into and out of stand-by mode (all subjects). Experienced users of dual site EMG choose to retain dual site control for one DOF (typically hand open/close, and then use IMU controls for other DOFs and functions.

Although the technological capabilities of the DEKA Arm promise increase functionality, limited research has compared functional abilities with the DEKA Arm to function with the conventional prostheses [5] and no studies have compared outcomes such as quality of life and community integration. One earlier study directly compared performance based and self-reported outcomes from 26 subjects using both the DEKA Arm and conventional prostheses. Subjects using either the Generation 2 (Gen 2) (N = 17) or Generation 3 (Gen 3) (N = 9) prototypes of the DEKA Arm[5] found that use of the prosthesis to perform activities, spontaneity of prosthesis use and perceived difficulty performing self-selected tasks was greater with the DEKA Arm as compared to conventional prosthesis, but dexterity scores were worse in 2/7 tests. However, differences in outcomes varied by configuration level; for example, for SC users’ activity performance was rated better when using the DEKA Arm as compared to the conventional prosthesis.

Conclusions based on the prior analyses are limited for several reasons. First, all subjects were experienced prosthesis users (experience ranging from 3 months to decades), and their exposure to the DEKA Arm was limited to structured training. Therefore the amount of exposure to each type of device was not equivalent. Despite structured prosthetic training in the study, some subjects may not have fully acclimated to the DEKA Arm. It is conceivable that with additional experience gained from home use their perceived function and functional performance would continue to improve. Another limitation of the earlier study is that it compared outcomes of the existing prosthesis to outcomes of both the Generation 2 (Gen 2) and Generation 3 (Gen 3) prototypes of the DEKA Arm and did not perform any sub-group analysis by device prototype. Given that the Gen 3 prototype included significant design changes meant to improve usability [1] it is possible that the inclusion of Gen 2 users biased comparisons of the DEKA Arm to the conventional prostheses towards the null. Lastly, the earlier analysis compared outcomes of self-reported disability, prosthesis use, and dexterity and activity performance but did not compare other important outcomes such as quality of life and community integration that theoretically could be impacted by upper limb prosthesis use.

Additional research is needed to build upon prior work. Therefore, the purposes of this study were to: 1) compare self-reported function, dexterity, activity performance, quality of life and community integration of the Gen 3 DEKA Arm to conventional prostheses; and 2) examine differences in outcomes by conventional prosthesis type, terminal device type and by DEKA Arm configuration level.

## Methods

### Study design

The VA Home Study of an Advanced Upper Limb Prosthesis (Home Study) was a quasi-experimental study that used a time series design. The research was approved by the Institutional Review Boards of the Providence VA Medical Center, the James Haley VA (Tampa), the VA NY Health Harbor System, and the Center for the Intrepid at Brooke Army Medical Center. Written informed consent was obtained from all subjects. The study had 2 parts: in-laboratory training with the DEKA Arm (Part A) and home use with the DEKA Arm (Part B). All participants enrolled in Part A and then a subset continued to Part B. During Part A participants were fit with and trained to use the DEKA Arm. During Part B participants used the DEKA Arm at home for up to 12 weeks, and returned for in-person re-evaluations on-site every 4 weeks.

### Subjects

Participants who reported that they regularly used a personal prosthesis (prosthesis users) and completed Part A of the study were included in this analysis. Participants eligible for Part A enrollment in the Home study were at least 18 years old and had an upper limb amputation at the transradial, transhumeral, shoulder disarticulation or scapulothoracic level. Persons with residual limb or skin conditions prohibiting socket fitting or with serious health conditions which the study staff believed would limit participation were excluded. At the End of Part A, the Principal Investigator in consultation with the study staff determined the eligibility for Part B based on observed behavior during Part A. Participants were eligible if they had at least fair functional use of the DEKA Arm (as gauged by the study occupational therapist), and demonstrated consistent safety awareness and sound judgement and the ability to troubleshoot minor technical issues.

### Data collection

At Baseline, at the End of Parts A and B, the study occupational therapists (OTs) administered a set of standardized measures to participants (Table 1). Participants, who were prosthesis users, usually completed performance measures at Baseline wearing their own prosthesis. However, on occasion, a prosthesis user did not utilize his/her device during Baseline testing because it was unavailable or broken. At Baseline, questions in self-report measures referred only to the participant’s personal prosthesis. At the End of Parts A and B, participants answered questions pertaining to the DEKA Arm and completed performance tests using the DEKA Arm.

**Table 1:** Outcome measures.

Measure	Construct	Brief description	Response	Higher scores indicate…
**Dexterity**				
Jebsen-Taylor Hand Function Test (JTHF)	Dexterity	7 tests of hand function	Performance speed; items.sec	better performance
**Activity**				
Activities Measure for Upper-Limb Amputees (AM-ULA)	Activity performance	18-everyday tasks	Task completion: speed, movement quality, skill and independence	better performance
University of New Brunswick Test of Prosthetic Function (UNB): Skill	Prosthetic skill	10 components of daily tasks requiring bimanual engagement	Skillfulness of terminal device use.	better performance
University of New Brunswick Test of Prosthetic Function (UNB): Spontaneity	Prosthetic spontaneity	10 components of daily tasks requiring bimanual engagement	Spontaneity of engaging the prosthesis in activities	better performance
Timed Measure of Activity Performance (T-MAP)	Activity performance	5 activities of daily living	Task completion: speed	Worse performance
Brief Activity Measure for Upper Limb Amputees (BAM-ULA)	Activity performance	10 items of functional task performance	Task completion: Unable to complete; Can complete	better performance
**Self-Reported Function**				
Disabilities of the Arm, Shoulder and Hand Score (QuickDASH)	Disability	Self-reported functional difficulty (8 items) 3 items about sleep, sensation and pain	Performance difficulty and impairment severity	greater disability
Upper-Extremity Functional Scale (UEFS)	Activity performance	Self-reported difficulty performing 23 everyday activities	Difficulty in performance	greater difficulty
Upper-Extremity Functional Scale (Use)	Use of prosthesis	Self-reported use of the prosthesis during everyday activities	Prosthesis use	more activities done with prosthesis
Patient-Specific Functional Scale (PSFS)	Difficulty performing activities	5 self-selected activities difficult to do because of the amputation	Difficulty in performance	less difficulty
**Other Measures**				
Wong-Baker FACES Pain Rating Scale	Pain	Six faces showing levels of pain severity	Pain intensity	more pain
Quality of Life (QOL)	Quality of life	16 question items about quality of life	Satisfaction with quality of life	better QOL
The Community Reintegration of Service Members Computer Adaptive test (CRIS-CAT)		Computer adaptive testing measuring participation in life roles		better community integration
CRIS-CAT Extent of Participation	Extent of participation		Frequency and amount	
CRIS-CAT Perceived Limitations	Perceived difficulty		Perceived limitations	
CRIS-CAT Satisfaction with Participation	Satisfaction		Satisfaction scale	
Trinity Amputation and Prosthesis Experience Scales (TAPES)	Prosthetic satisfaction	10 items satisfaction with prosthesis	Satisfaction	greater satisfaction

We selected a broad range of validated measures to assess important constructs for upper limb amputees. Performance based measures included a dexterity measure, theJebsen-Taylor Hand Function Test (JTHFT),[6, 7] 4 and measures of activty performance: the Activities Measure for Upper Limb Amputees (AM-ULA) [8]; University of New Brunswick Test of Prosthetic Function for Unilateral Amputees (UNB);[9, 10] Timed Measure of Activity Performance (T-MAP), [11] and Brief Activity Measure for Upper Limb Amputees (BAM-ULA).[12] Although each of the activity measures assesses performance of daily activities, they differ considerably in their scoring criteria and item content. For instance, the T-MAP assesses the time it takes to perform an activity; while the AM-ULA assesses body compensation during activity performance. Given that there is no accepted gold standard activity measure, we believed that inclusion of multiple metrics would provide important information. Self-report Measures included: Disabilities of the Arm, Shoulder and Hand Score (QuickDASH),[13] Upper Extremity Functional Scale (UEFS),[14] Patient Specific Functional Scale (PSFS),[15] Wong-Baker FACES Pain Rating Scale (Wong-Baker),[16] Quality of Life (QOL) scale,[17] Community Reintegration of Service Members Computer Adaptive test (CRIS-CAT),[18] and; Trinity Amputation and Prosthesis Experience Satisfaction Scale (TAPES).[19] Each measure is described below.

#### Modified Jebsen-Taylor Hand Function (JTHFT)

The JJTHF is a measure of dexterity and simple functional activities[20]. The 7 JTHF subtasks are: writing; page turning; lifting small objects; feeding; lifting large, lightweight objects; lifting large, heavy objects; stacking checkers. The modified version used in this study caps maximal allowable time for each subtask at 2 minutes and scores the number of items completed per second. Thus, higher scores indicate better performance. The reliability and validity of the modified version was demonstrated in upper limb amputees[21], and the responsiveness of specific sub-tasks to prosthetic training with the DEKA Arm has been reported. [22]

#### The Activities Measure for Upper-Limb Amputees (AM-ULA)

The AM-ULA is measure of activity performance for prosthesis users.[8] The test has 18 items, each of which is scored from 0–4 (unable to excellent), with higher scores indicating better functional performance. The scoring rubric takes a variety of aspects of activity performance into consideration including: sub-task completion, skillfulness of prosthesis use, movement quality, independence, and overall time to perform that activity. Analysis of psychometric properties showed that it has excellent test-retest reliability, interrater reliability, and internal consistency and demonstrated known group validity[8]. The AM-ULA was been shown to be responsive to change after prosthetic training. [22]

#### University of New Brunswick test of prosthetic function (UNB)

The UNB test is a measure of prosthetic skill and spontaneity that is appropriate for unilateral amputees. [9]. The spontaneity scale measures the extent to which the amputee has incorporated the prosthesis into his or her body image and measures the tendency to use the prosthesis to assist with the task. The skill scale measures the dexterity with which the prosthesis is used. It includes the ability to open and close the terminal device to grasp and release objects with confidence, speed and consistency and maintain grasp without letting go accidentally, and apply correct amount of pressure. This study used one of the subtests of activities designed for ages 11–13 year olds which included 10 activities related to (1) wrapping a parcel, (2) sewing a button on cloth, (3) cutting meat, (4) drying dishes, and (5) sweeping floors. Each activity is rated a 5-point scale of 0–4 for dual functions: spontaneity of prosthetic function (Spontaneity) and skill of prosthetic function (Skill). Higher UNB scores indicate better performance. This subtest has been found to have acceptable internal consistency, test-retest, and interrater reliability and evidence of validity[23], and was responsive to change after prosthetic training. [22]

#### Timed Measure of Activity Performance (T-MAP)

The T-MAP is a timed based measure of common activity performance developed for upper limb amputees. [11]. It consists of 5 items that were adapted from the Rivermead Extended Activities Index [24] an Instrumental Activities of Daily Living (IADL) measure. The items in the T-MAP are: (1) drink, (2) wash face, (3) food preparation, (4) eating, and (5) dressing activities. The T-MAP revealed has very good internal consistency, excellent test-retest reliability, and evidence of construct validity [11].

#### Brief Measure of Activity performance (BAM-ULA)

The BAM-ULA is an observational measure of activity performance [12]. The 10 items included in the measure are: tuck a shirt in pants; lift a 20 lb. bag; open a water bottle; remove a wallet from back pocket; replace the wallet in back pocket; take a gallon of water from the refrigerator and place on the counter (lift gallon jug); pour water from a gallon jug; brush or comb hair; use a fork; and open a door with knob. Each item is rated 0 for ‘unable to complete’, or 1 for ‘did complete’. The scores for each item are summed to obtain the overall task completion score. Summary scores are calculated only when all 10 items are rated. Higher task completion scores indicated better performance. Analyses of psychometric properties in a sample of persons with upper limb amputation showed that the BAM-ULA has acceptable internal consistency, test-retest reliability, and displays evidence of construct and concurrent validity [12].

#### QuickDASH

The QuickDASH is an 11-item disability scale, a shorter version of the Disabilities of the Arm and Shoulder (DASH) measure, which has been validated for use in upper limb amputation. [13] It includes 8 items related to difficulty performing functional activities and 3 items level severity of impairments[25]. Respondents indicate the amount of difficulty performing activities, amount of limitation, or the extent of interference with activities (using 1–5 Likert scales with 1 indicating the least impairment and 5 indicating the most). Items asking about extent of arm, shoulder and hand pain and tingling are rated from 1 (none) to 5 (extreme). The QuickDASH has strong internal consistency and test-retest reliability in upper limb amputees, and demonstrates evidence of known group and construct validity. Additionally, the QuickDASH was shown to be moderately responsive to prosthetic training. [13]

#### The Upper-Extremity Functional Scale (UEFS)

Upper Extremity Functional Scale (UEFS) is one of the scales of the Orthotics and Prosthetics Users Survey (OPUS). [10, 14] The OPUS UEFS is the only self-report measure of activity performance developed specifically for adults with upper limb amputation.[26] It is a self-report measure of difficulty performing and ease of performing 23 every day activities self-care and IADL tasks [10, 14]. The tasks are rated on a 1–5 point scale (very easy to cannot perform activity), regardless of how the activities are performed (with or without a prosthesis). Total score are calculated using IRT methods. Higher scores indicate more difficulty in performing activities. However, respondents also indicate which of the items were performed using a prosthesis. These responses are used in the UEFS Use scale which is the proportion of items performed using the prosthesis. Higher scores of the UEFS Use scale indicate that more activities are done with a prosthesis. This study used 22 of the 23 UEFS items from the original measure. [5], eliminating the item “washing face”. The modified UEFS and UEF use scales have been shown to be reliable[8]. However, the UEFS, did not differentiate amputees users by level of amputation[21] and was not responsive to change after prosthesis training.,[27]

#### The Patient Specific Functional Scale (PSFS)

The PSFS is a patient-specific outcome measure that assesses functional status. The PSFS asks patients to identify up to five activities that they have difficulty performing due to their condition and then rate the amount of limitation they have in performing these activities on a scale of 0 to 10, with 0 being unable to perform the activity and 10 being able to perform the activity with no problem. Individual items are scored separately. Validity of the PSFS in a sample with upper-limb amputation was supported. [8] The PSFS was reported to be responsive to change for patients with arm impairments,[28] and for those with upper limb amputation who participate in prosthetic training with the DEKA Arm. [22]

#### The Wong-Baker FACES Pain Rating Scale (FACES)

The Wong-Baker FACES is a self-report measure of pain[29]. This measure has a 6-point pain scale that utilizes faces to indicate different levels of pain intensity. Patients are asked to choose the face that best describes how he/she is feeling. Higher pain scores indicate more severe pain

#### Quality of Life Scale (QOL)

The QOL consists of 16 questions that assess satisfaction with a variety domains that diverse patient groups with chronic illness define as quality of life [17]. Its items address: material comforts, health, relationships with family, intimate relationships, friendships, childrearing, helping others, participating in organizations, learning, self-knowledge, working, self-expression, socializing, being entertained, participating in active recreations and being independent. Patients are asked to describe how satisfied they are using a 1–7 scale (Terrible to Delighted). Higher scores indicate better quality of life. Reliability of the QOL scale in patients with a variety of chronic illnesses is supported. [30]

#### The Community Reintegration of Service Members Computer Adaptive test (CRIS-CAT)

The CRIS-CAT is a computer adaptive test version of the CRIS measure. [18,31] Like the CRIS, the CRIS-CAT has three sub-scales, each is comprised of items drawn from the 9 activity and participation content domains (or chapters), defined by the ICF. The Perceived Limitations to Participation subscale assesses Veterans’ perceived limitations in participation. The Extent of Participation subscale assesses how often Veterans experience a challenge in participation. The Satisfaction with Participation subscale assesses Veterans’ level of satisfaction with participation. Higher scores indicate better community integration. Reliability, structural, concurrent, construct and predictive validity of the CRIS-CAT scales have been reported. [32, 33]. Higher scores indicate better community integration

#### Trinity Amputations and Prosthetics Experience Scale (TAPES) Satisfaction Scale

The TAPES is a condition-specific instrument that assesses the psychosocial processes involved in adjusting to a prosthesis, the specific demands of wearing a prosthesis and the potential sources of maladjustment. The TAPES contains individually scored subscales, divided into 3 sections (psychosocial scales, activity restriction, and satisfaction with prosthesis).[34, 35] For device satisfaction, respondents to the TAPES indicate their level of satisfaction on a 5-point scale (very dissatisfied to very satisfied) regarding 10 items: color, shape, noise, appearance, weight, usefulness, reliability, fit, comfort and overall satisfaction. Higher scores indicate greater satisfaction. The prosthetic satisfaction scale has been shown to have excellent internal consistency for upper limb amputees[19].

### Data analysis

Participant demographics were examined for all prosthesis users who completed Part A as well as for the subgroup of prosthesis users who completed Part B. Descriptive statistics of all performance-based and self-report measures were examined at Baseline, End of Part A, and End of Part B. Scores for performance-based and self-report measures were compared between Baseline and End of A using paired t-tests. Wilcoxon signed-rank tests were used to compare Baseline and End of B outcomes for the full sample, and for the sample stratified by device type (body-powered or myoelectric) and terminal device type (single or multi-degree of freedom). Descriptive statistics of all measures by configuration level of the DEKA Arm were also compared graphically at Baseline, End of A and End of B. We also calculated Effect sizes (ES) differences for the full sample to quantify the magnitude of differences for those tests that were found to be statistically significantly different.

Multiple categories were identified to adjust for false discovery rates in “families” or categories of tests. The following categories were used: dexterity (7 measures), activity performance (5 measures), self-reported function (4 measures), pain, quality of life and community integration, satisfaction with prosthesis (6 measures). The Benjamini-Hochberg method was used to maintain a false discovery rate of 0.10 within each category of tests.

## Results

Characteristics of 23 prosthesis users (mean age = 45 ± 16; 87% male) who completed Part A and the 15 prosthesis users (mean age = 45 ± 18; 87% male) who completed Parts A and B are shown in Table 2. Fifty two percent of Part A completers used an RC DEKA Arm, 30.4% used an HC Arm and 17.4% used an SC. Amongst Part B completers 53.3% used an RC, 33.3% an HC and 13.3% SC. The majority of participants used a myoelectric, single degree of freedom (DOF) prosthesis at Baseline (Table 2).

**Table 2:** Subject characteristics at each testing point (N = 23).

	Completed A	Completed B
	N = 23	N = 15
	Mn (sd)	Mn (sd)
**Age**	45.3 (16.0)	44.6 (17.6)
**Months of prosthesis Use**	182.9 (195.8)	167.9 (196.6)
	**N(%)**	**N(%)**
**Gender**		
Male	20 (87.0)	13 (86.7)
Female	3 (13.0)	2 (13.3)
**Race**		
White only	20 (87.0)	13 (86.7)
Black only	3 (13.0)	2 (13.3)
Mixed/other	0 (0.0)	0 (0.0)
**Veteran Status**		
Non-Veteran	8 (34.8)	5 (33.3)
Veteran	11 (47.8)	7 (46.7)
Active Duty	4 (17.4)	3 (20.0)
**Amputation Level**		
Transradial	12 (52.2)	8 (53.3)
Transhumeral	9 (39.1)	6 (40.0)
Shoulder disarticulation/forequarter	2 (8.7)	1 (6.7)
**DEKA Arm Configuration Level**		
RC	12 (52.2)	8 (53.3)
HC	7 (30.4)	5 (33.3)
SC	4 (17.4)	2 (13.3)
**Control Scheme**		
IMU + other control* for mode/standby	4(17.39)	0
IMU + EMG + other control* for mode/standby	17(73.91)	14 (93.33)
IMU + EMG	1(4.35)	1 (6.67)
EMG + other*control for mode/standby	1(4.35)	0
**Prosthesis Used at Baseline**		
Body Powered	7 (30.4)	5 (33.3)
Myoelectric	15 (65.2)	10 (66.7)
Hybrid	1 (4.4)	0 (0.0)
**Terminal device type**		
Single degree of freedom	17 (73.9)	11 (73.3)
Multiple degree of freedom	6 (26.1)	4 (26.7)

*Other controls may include pressure transducer, linear transducer, or rocker switch for mode selection. The majority used pressure transducer.

Table 3 shows scores for all measures by testing period. T-MAP time to completion was shorter at Baseline as compared to the End of A (P<0.001), but comparable between Baseline and End of B. AM-ULA scores improved (P<0.005) from Baseline to End of B. There were no significant changes between Baseline and End of A or End of B in any other measure of dexterity or activity performance by testing period.

**Table 3:** Outcomes across assessment time points.

		Baseline	End of A	T test			Baseline	End of B	W S-R
	N	Mn (sd)	Mn (sd)	P	N	Mn (sd)	Mn (sd)	P
**Dexterity**								
Jebsen-Taylor Hand Function (JTHFT)								
JTHFT: Writing items/sec	23	0.33 (0.23)	0.34 (0.14)	0.9284	15	0.35 (0.25)	0.45 (0.20)	0.1354
JTHFT: Page Turning items/sec	23	0.07 (0.07)	0.06 (0.04)	0.2496	14	0.10 (0.07)	0.11 (0.07)	1.0000
JTHFT: Small items items/sec	23	0.07 (0.08)	0.08 (0.08)	0.3730	14	0.08 (0.09)	0.09 (0.07)	0.6698
JTHFT: Feeding / Eating items/sec	23	0.10 (0.08)	0.07 (0.05)	0.1164	14	0.12 (0.08)	0.08 (0.08)	0.0785
JTHFT: Checkers items/sec	23	0.09 (0.08)	0.08 (0.07)	0.6318	14	0.09 (0.08)	0.11 (0.08)	0.1937
JTHFT: Light Cans items/sec	23	0.20 (0.13)	0.20 (0.16)	0.7603	14	0.22 (0.14)	0.26 (0.18)	0.2958
JTHFT: Heavy Cans items/sec	23	0.22 (0.14)	0.21 (0.17)	0.9105	14	0.24 (0.14)	0.29 (0.16)	0.3575
**Activity**								
AM-ULA	23	16.7 (5.4)	17.1 (4.8)	0.5851	13	16.5 (4.8)	19.8 (4.5)	*0.0024
UNB: Spontaneity	22	3.1 (0.5)	3.1 (0.5)	0.9610	13	3.1 (0.4)	3.3 (0.4)	0.1943
UNB: Skill	22	2.9 (0.5)	2.9 (0.5)	0.9038	13	3.0 (0.5)	3.2 (0.4)	0.1138
T-MAP	20	533.8(228.5)	786.6(413.1)	*0.0008	11	508.6 (264.2)	676.6 (469.6)	0.3203
BAM-ULA summary (new)	16	6.9 (3.0)	7.5 (1.7)	0.3002	10	7.7 (2.2)	8.3 (1.5)	0.6563
**Self-reported function**								
QuickDASH	23	28.3 (13.0)	21.9 (10.3)	*0.0108	15	26.5 (11.3)	20.8 (12.0)	*0.0313
Upper Extremity Functional Scale (UEFS)	14	44.4 (6.0)	44.2 (4.5)	0.8810	10	43.0 (5.4)	38.6 (9.3)	0.2754
UEFS use	23	0.4 (0.2)	0.7 (0.3)	*0.0060	14	0.5 (0.2)	0.7 (0.2)	*0.0105
Patient Specific Functional Scale (PSFS)	23	2.6 (1.4)	5.3 (1.8)	*0.0001	15	2.6 (1.3)	6.2 (2.0)	*0.0001
**Quality of life etc.**								
Wong-Baker Pain Scale	23	0.8 (1.0)	0.9 (1.1)	0.7040	15	0.5 (0.7)	0.9 (1.0)	**0.0313**
Quality of Life (QOL) Scale	23	5.7 (0.6)	5.7 (0.7)	0.9201	15	5.7 (0.6)	5.8 (0.8)	0.5526
Community integration								
CRIS-CAT Extent of Limitations	22	54.4(9.2)	54.9 (8.5)	0.7853	15	54.5 (9.2)	57.6 (10.1)	0.2378
CRIS-CAT Perceived Limitations	22	55.9 (14.6)	51.5 (9.4)	0.2001	15	57.5 (17.0)	60.3 (19.2)	0.4810
CRIS-CAT Satisfaction with Participation	22	53.4 (12.3)	50.7 (5.9)	0.2182	15	54.9 (14.0)	56.0 (13.9)	0.6721
TAPES Satisfaction Scale	23	3.5 (0.6)	3.5 (0.7)	0.8222	15	3.6 (0.6)	3.7 (0.9)	0.5151

*significant after Benjamini-Hochberg adjustment with false discovery rate = 0.1.

Outcomes for three self-reported measures of function, the QuickDASH, UEFS use scale and PSFS, improved from Baseline to End of A and from Baseline to the End of B. Wong Baker Pain Scale ratings increased from Baseline to End of B but this finding was no longer statistically significant after correcting for multiple comparisons with the Benjamini-Hochberg procedure. All other statistically significant findings remained significant after correcting for multiple comparisons. Table 4 shows the ES for each of the statistically significant results.

**Table 4:** Effect size calculations for tests that were significantly different at Baseline vs. End of A and Baseline vs. End of B.

	End of A	End of B
	ES	ES
**Performance Measures**		
AM-ULA	NS*	0.71
T-MAP	0.76	NS
**Self-reported function**		
QuickDASH	0.55	0.49
UEFS use	1.18	1.00
Patient Specific Functional Scale (PSFS)	1.67	2.13

*NS not a statistically significantly difference.

Table 5 shows the outcomes across testing periods by type of conventional prosthesis used at Baseline. For the myoelectric/hybrid device users, the pattern of results comparing Baseline and End of A were similar to findings in the full sample (improved QuickDASH, UEFS use and PSFS scores and worse T-MAP). For body powered users, the JTHFT page turning test score was lower and T-MAP scores were worse (P < .05), but did not remain statistically significant after correcting for multiple comparisons. From Baseline to End of B the pattern of results were similar to the overall group for myoelectric prosthesis users; however, after correcting for multiple comparisons only the improvement in PSFS scores remained statistically significant. Among body-powered users, there were no statistically significant differences between Baseline and End of B.

**Table 5:** Outcomes across assessment time points by device type at Baseline and End of A / End of B.

	Baseline (BL) to End of A (EOA)						Baseline (BL) to End of B (EOB)					
	Body Powered (N = 7)			Myoelectric/Hybrid (N = 16)			Body Powered (N = 5)			Myoelectric/Hybrid (N = 10)		
	BL	EOA	W S-R	BL	EOA	W S-R	BL	EOB	W S-R	BL	EOB	W S-R
	Mn (sd)	Mn (sd)	P	Mn (sd)	Mn (sd)	P	Mn (sd)	Mn (sd)	P	Mn (sd)	Mn (sd)	P
**Dexterity**												
Jebsen-Taylor Hand Function (JTHFT) items/sec												
JTHFT: Writing	0.35 (0.26)	0.33 (0.17)	0.938	0.32 (0.22)	0.34 (0.14)	0.860	0.36 (0.28)	0.33 (0.19)	1.000	0.34 (0.24)	0.50 (0.19)	**0.049**
JTHFT: Page Turning	0.11 (0.08)	0.04 (0.02)	**0.031**	0.06 (0.06)	0.06 (0.04)	0.413	0.12 (0.08)	0.08 (0.05)	0.313	0.08 (0.06)	0.12 (0.08)	0.426
JTHFT: Small items	0.07 (0.07)	0.06 (0.03)	0.688	0.06 (0.08)	0.10 (0.09)	0.309	0.07 (0.08)	0.06 (0.03)	1.000	0.08 (0.10)	0.11 (0.08)	0.652
JTHFT: Feeding / Eating	0.13 (0.10)	0.09 (0.06)	0.219	0.09 (0.06)	0.07 (0.05)	0.274	0.17 (0.10)	0.07 (0.06)	0.063	0.09 (0.05)	0.08 (0.09)	0.496
JTHFT: Checkers	0.05 (0.04)	0.06 (0.05)	0.297	0.11 (0.09)	0.09 (0.07)	0.433	0.06 (0.05)	0.10 (0.09)	0.125	0.10 (0.09)	0.11 (0.07)	0.652
JTHFT: Light Cans	0.10 (0.06)	0.11 (0.08)	0.813	0.24 (0.13)	0.24 (0.17)	0.706	0.11 (0.06)	0.15 (0.07)	0.438	0.28 (0.13)	0.32 (0.19)	0.652
JTHFT: Heavy Cans	0.12 (0.10)	0.11 (0.07)	0.938	0.26 (0.14)	0.26 (0.18)	0.940	0.13 (0.10)	0.18 (0.09)	0.625	0.31 (0.13)	0.34 (0.16)	0.496
**Activity**												
AM-ULA	14.5 (4.6)	14.7 (4.9)	0.938	17.6 (5.5)	18.1 (4.5)	0.678	15.4 (5.0)	18.7 (3.1)	0.063	17.2 (4.8)	20.6 (5.3)	**0.047**
UNB: Spontaneity	3.1 (0.4)	3.0 (0.3)	0.469	3.1 (0,5)	3.1 (0.5)	0.652	3.2 (0.3)	3.3 (0.4)	0.813	3.0 (0.5)	3.3 (0.4)	0.156
UNB: Skill	3.0 (0.5)	2.7 (0.3)	0.344	2.9 (0.5)	3.0 (0.6)	0.302	3.1 (0.5)	3.2 (0.4)	1.000	2.9 (0.5)	3.2 (0.4)	**0.031**
T-MAP	469.3 (94.0)	827.0 (354.9)	**0.031**	568.5 (272.9)	764.9 (462.3)	*0.013	472.8 (105.1)	880.5 (501.5)	0.125	529.0 (330.9)	560.1 (445.4)	0.938
BAM-ULA summary (new)	4.8 (3.1)	6.8 (0.8)	0.250	7.8 (2.5)	7.8 (1.9)	1.000	6.7 (2.3)	9.0 (1.0)	0.500	8.1 (2.1)	8.0 (1.6)	1.000
**Self-reported function**												
QuickDASH	36.7 (15.6)	26.3 (8.3)	0.156	24.6 (10.1)	20.0 (10.7)	*0.043	32.7 (13.7)	25.0 (12.9)	0.313	23.4 (9.0)	18.6 (11.6)	0.131
Upper Extremity Functional Scale (UEFS)	46.0 (6.9)	43.2 (6.0)	0.563	42.8 (5.0)	45.1 (2.3)	0.156	43.4 (5.6)	42.2 (7.6)	1.000	42.5 (5.8)	35.1 (10.3)	0.125
UEFS use	0.5 (0.2)	0.7 (0.2)	0.125	0.4 (0.2)	0.6 (0.3)	*0.049	0.5 (0.2)	0.6 (0.2)	0.250	0.5 (0.2)	0.7 (0.2)	0.055
Patient Specific Functional Scale (PSFS)	2.5 (1.1)	6.1 (1.3)	**0.031**	2.7 (1.5)	4.9 (1.8)	*0.001	3.0 (0.9)	6.7 (1.7)	0.063	2.4 (1.5)	6.0 (2.2)	*0.002
**Quality of life etc.**												
Wong-Baker Pain Scale	1.6 (1.3)	1.1 (0.7)	0.438	0.5 (0.6)	0.8 (1.2)	0.258	1.0 (1.0)	1.6 (1.3)	0.250	0.3 (0.5)	0.6 (0.7)	0.250
Quality of Life (QOL) Scale	5.8 (0.7)	6.1 (0.7)	0.234	5.6 (0.5)	5.5 (0.6)	0.571	6.0 (0.8)	6.1 (0.8)	0.438	5.6 (0.4)	5.6 (0.8)	0.910
Community integration CRIS-CAT												
Extent of Limitations	55.5 (10.7)	55.5 (4.3)	0.875	54.0 (8.9)	54.6 (9.7)	0.845	54.0 (11.3)	58.6 (7.2)	0.250	54.8 (8.7)	57.1 (11.6)	0.633
Perceived Limitations	60.3 (17.8)	51.7 (8.5)	0.219	54.2 (13.6)	51.4 (10.0)	0.465	62.4 (18.8)	62.4 (16.3)	1.000	55.1 (16.5)	59.3 (21.2)	0.375
Satisfaction with Participation	58.7 (13.1)	52.8 (6.9)	0.156	51.4 (11.8)	49.9 (5.5)	0.688	60.0 (14.1)	56.6 (11.7)	0.250	52.4 (14.0)	55.7 (15.4)	0.152
TAPES Satisfaction Scale	3.4 (0.6)	3.8 (0.8)	0.375	3.6 (0.6)	3.4 (0.7)	0.131	3.5 (0.6)	4.0 (0.9)	0.438	3.5 (0.5)	3.6 (0.9)	0.922

*significant after Benjamini-Hochberg adjustment with false discovery rate = 0.1.

Table 6 shows scores for all outcomes across testing periods by degrees of freedom of the conventional prosthesis terminal device. Comparisons between Baseline and End of A showed that, users of a single DOF device had significantly worse JTHFT Feeding and T-MAP scores, but improved UEFS use and PSFS scores. In contrast, the only statistically significant difference in outcomes for multi-degree of freedom device users was in the JTHF feeding score which improved, however this was no longer statistically significant after controlling for multiple comparisons. Comparisons between Baseline and End of B showed that users of single DOF devices had worse JTHF feeding scores but improved AM-ULA, QuickDASH and PSFS scores, while no statistically significant differences were observed in users of multi DOF devices.

**Table 6:** Outcomes across assessment time points by device type at Baseline and End of A / End of B.

	Baseline (BL) to End of A (EOA)						Baseline (BL) to End of B (EOB)					
	Single DOF (N = 17)			Multi DOF (N = 6)			Single DOF (N = 11)			Multi DOF (N = 4)		
	BL	EOA	W S-R	BL	EOA	W S-R	BL	EOB	W S-R	BL	EOB	W S-R
	Mn (sd)	Mn (sd)	P	Mn (sd)	Mn (sd)	P	Mn (sd)	Mn (sd)	P	Mn (sd)	Mn (sd)	P
**Dexterity**												
Jebsen-Taylor Hand Function (JTHFT) items/sec												
JTHFT: Writing	0.36 (0.24)	0.32 (0.14)	0.306	0.27 (0.20)	0.38 (0.16)	0.156	0.34 (0.28)	0.39 (0.18)	0.465	0.35 (0.17)	0.60 (0.18)	0.250
JTHFT: Page Turning	0.08 (0.07)	0.05 (0.04)	0.129	0.06 (0.06)	0.08 (0.04)	0.438	0.10 (0.07)	0.08 (0.04)	0.275	0.08 (0.06)	0.18 (0.09)	0.375
JTHFT: Small items	0.07 (0.08)	0.09 (0.09)	0.730	0.06 (0.08)	0.08 (0.05)	0.688	0.09 (0.09)	0.08 (0.07)	0.770	0.06 (0.09)	0.11 (0.05)	0.625
JTHFT: Feeding / Eating	0.13 (0.08)	0.06 (0.05)	*0.001	0.04 (0.04)	0.11 (0.05)	**0.031**	0.14 (0.07)	0.07 (0.06)	*0.004	0.06 (0.04)	0.11 (0.12)	0.625
JTHFT: Checkers	0.09 (0.08)	0.07 (0.06)	0.080	0.09 (0.10)	0.12 (0.09)	0.844	0.08 (0.08)	0.11 (0.08)	0.232	0.09 (0.10)	0.11 (0.08)	0.875
JTHFT: Light Cans	0.16 (0.10)	0.18 (0.15)	0.352	0.29 (0.17)	0.26 (0.19)	0.844	0.16 (0.10)	0.19 (0.11)	0.160	0.36 (0.12)	0.41 (0.23)	0.625
JTHFT: Heavy Cans	0.19 (0.13)	0.17 (0.14)	0.548	0.30 (0.15)	0.32 (0.20)	0.563	0.19 (0.13)	0.22 (0.11)	0.625	0.37 (0.11)	0.46 (0.13)	0.375
**Activity**												
AM-ULA	16.1 (5.2)	16.1 (4.3)	0.917	18.2 (6.0)	19.9 (5.5)	0.438	15.7 (5.0)	18.8 (4.4)	*0.012	19.3 (3.3)	23.3 (3.5)	0.250
UNB: Spontaneity	3.1 (0.4)	3.0 (0.4)	0.295	2.9 (0.6)	3.1 (0.5)	0.563	3.2 (0.4)	3.4 (0.4)	0.367	3.0 (0.5)	3.2 (0.1)	0.500
UNB: Skill	3.0 (0.5)	2.9 (0.5)	0.511	2.7 (0.6)	3.0 (0.6)	0.313	3.1 (0.5)	3.2 (0.4)	0.367	2.7 (0.6)	3.0 (0.2)	0.250
T-MAP	547.1 (230.8)	824.1 (433.3)	*0.001	480.8 (244.0)	636.5 (369.5)	0.375	544.4 (294.1)	800.0 (494.9)	0.055	413.0 (165.4)	347.7 (149.5)	0.250
BAM-ULA summary (new)	6.6 (2.9)	7.3 (1.4)	0.445	7.6 (3.3)	8.0 (2.4)	0.750	7.0 (2.2)	8.0 (1.6)	0.500	9.3 (1.2)	9.0 (10)	1.000
**Self-reported function**												
QuickDASH	28.6 (14.0)	22.4 (10.4)	0.124	27.3 (10.9)	20.5 (10.8)	0.094	26.9 (11.9)	20.5 (13.3)	*0.043	25.6 (10.7)	21.6 (8.8)	0.625
Upper Extremity Functional Scale (UEFS)	44.7 (6.5)	44.0 (4.7)	0.966	42.6 (1.7)	44.9 (4.0)	0.500	42.9 (5.7)	39.7 (9.3)	0.496	43.8 (-)	29.6 (-)	-
UEFS use	0.5 (0.2)	0.7 (0.3)	*0.046	0.3 (0.2)	0.7 (0.4)	0.156	0.5 (0.2)	0.7 (0.2)	0.098	0.4 (0.1)	0.6 (0.1)	0.125
Patient Specific Functional Scale (PSFS)	2.6 (1.2)	5.1 (1.9)	*0.000	2.8 (1.8)	5.7 (1.1)	0.063	2.7 (1.1)	5.9 (2.3)	*0.001	2.3 (1.8)	7.0 (0.5)	0.125
**Quality of life etc.**												
Wong-Baker Pain Scale	0.9 (1.1)	1.1 (1.2)	0.681	0.5 (0.5)	0.3 (0.5)	1.000	0.6 (0.8)	1.1 (1.1)	0.063	0.3 (0.5)	0.5 (0.6)	1.000
Quality of Life (QOL) Scale	5.6 (0.6)	5.8 (0.7)	0.199	5.8 (0.4)	5.3 (0.5)	0.063	5.7 (0.6)	5.8 (5.9)	0.534	5.7 (0.4)	5.7 (0.6)	1.000
Community integration CRIS-CAT												
Extent of Limitations	53.3 (10.0)	54.1 (7.7)	0.901	57.5 (6.1)	56.8 (10.9)	0.750	53.5 (10.2)	57.6 (9.0)	0.160	57.3 (6.2)	57.5 (14.5)	1.000
Perceived Limitations	57.1 (16.7)	50.6 (7.2)	0.157	52.5 (6.5)	54.0 (14.5)	0.875	59.3 (19.3)	60.2 (17.8)	0.504	52.8 (8.3)	60.8 (25.7)	0.750
Satisfaction with Participation	54.6 (13.9)	50.5 (5.9)	0.239	50.2 (6.2)	51. 3 (6.5)	0.563	57.0 (15.6)	56.8 (14.3)	0.871	49.3 (7.4)	53.8 (14.4)	0.625
TAPES Satisfaction Scale	3.6 (0.6)	3.6 (0.8)	0.738	3.4 (0.4)	3.3 (0.4)	0.875	3.6 (0.6)	3.8 (1.0)	0.621	3.4 (0.5)	3.5 (0.5)	0.875

*significant after Benjamini-Hochberg adjustment with false discovery rate = 0.1.

**Fig 1:**
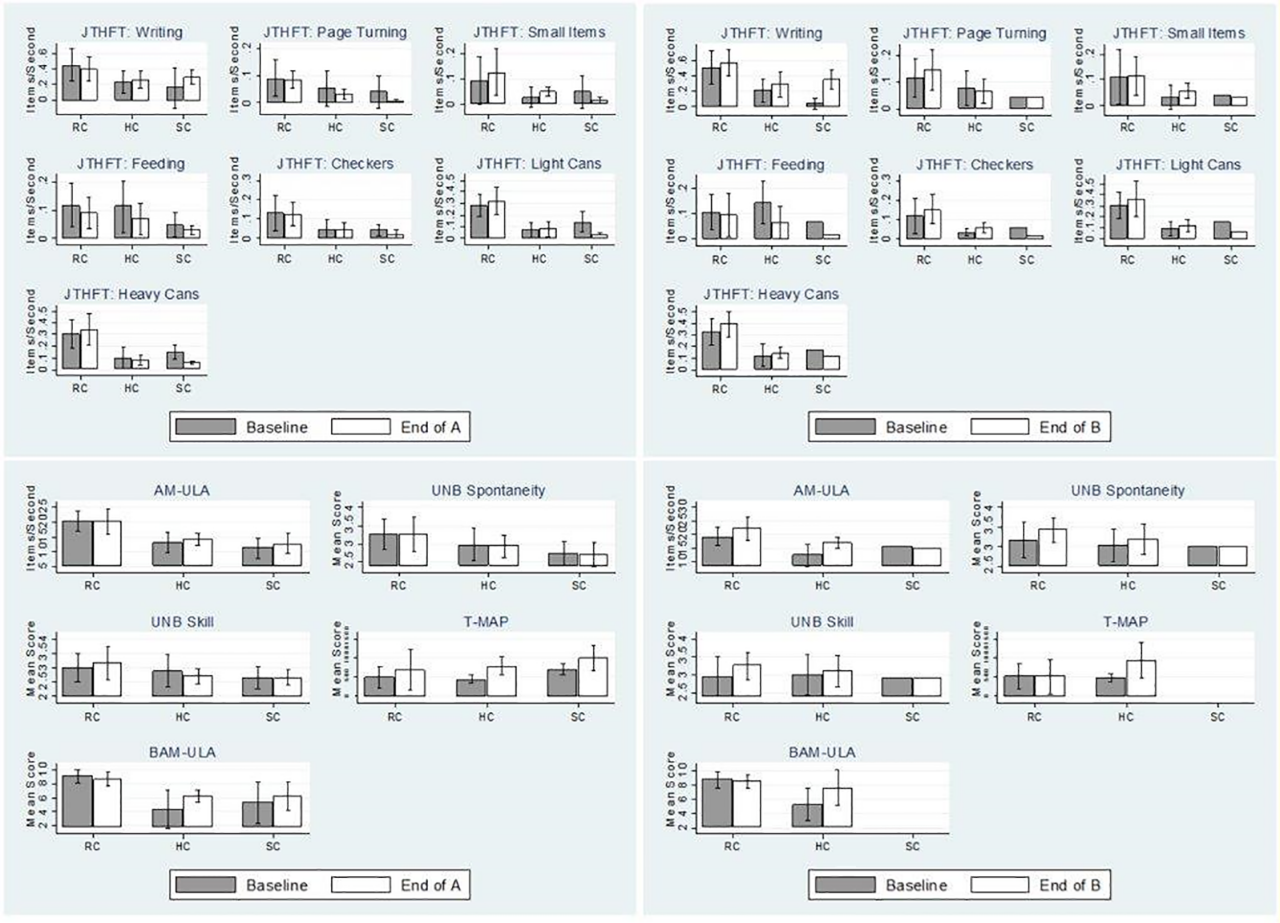
Performance-based measures at Baseline compared to End of A and End of B by configuration level.

Fig 1A–1D shows performance outcomes from Baseline to End of A and Baseline to End of B by DEKA Arm configuration level. Change in dexterity scores varied by JTHF item and DEKA level. At End of A, subjects using the RC and HC devices had improved scores for three JTHF items while SC users only improved on the writing item. From Baseline to End of Part B, dexterity scores improved for RC users on 6 items and for HC users on 5 items. SC users scores at End of B had only improved for the JTHF writing task.

AM-ULA scores were comparable for RC users at Baseline and End of A but better for HC and SC users. Comparisons of Baseline and End of B scores show AM-ULA scores better for RC and HC users, but equivalent for SC users. UNB Spontaneity scores were similar at Baseline and End of B, but improved at End of B for RC and HC users. UNB Skill scores improved for RC users at the End of A, declined for HC users and were equivalent for SC users. At the End of B UNB skill scores had improved slightly for HC users as well. From Baseline to End of A, T-MAP scores were greater (indicating slower performance) for all configuration levels. Whereas in the Baseline to End of B comparison T- MAP scores for the RC users were comparable, and scores for HC users still greater. From Baseline to End of A BAM-ULA scores were nearly equivalent for RC users, but improved for HC and SC users. This pattern persisted at End of B for RC and HC users (data not available for SC).

**Fig 2:**
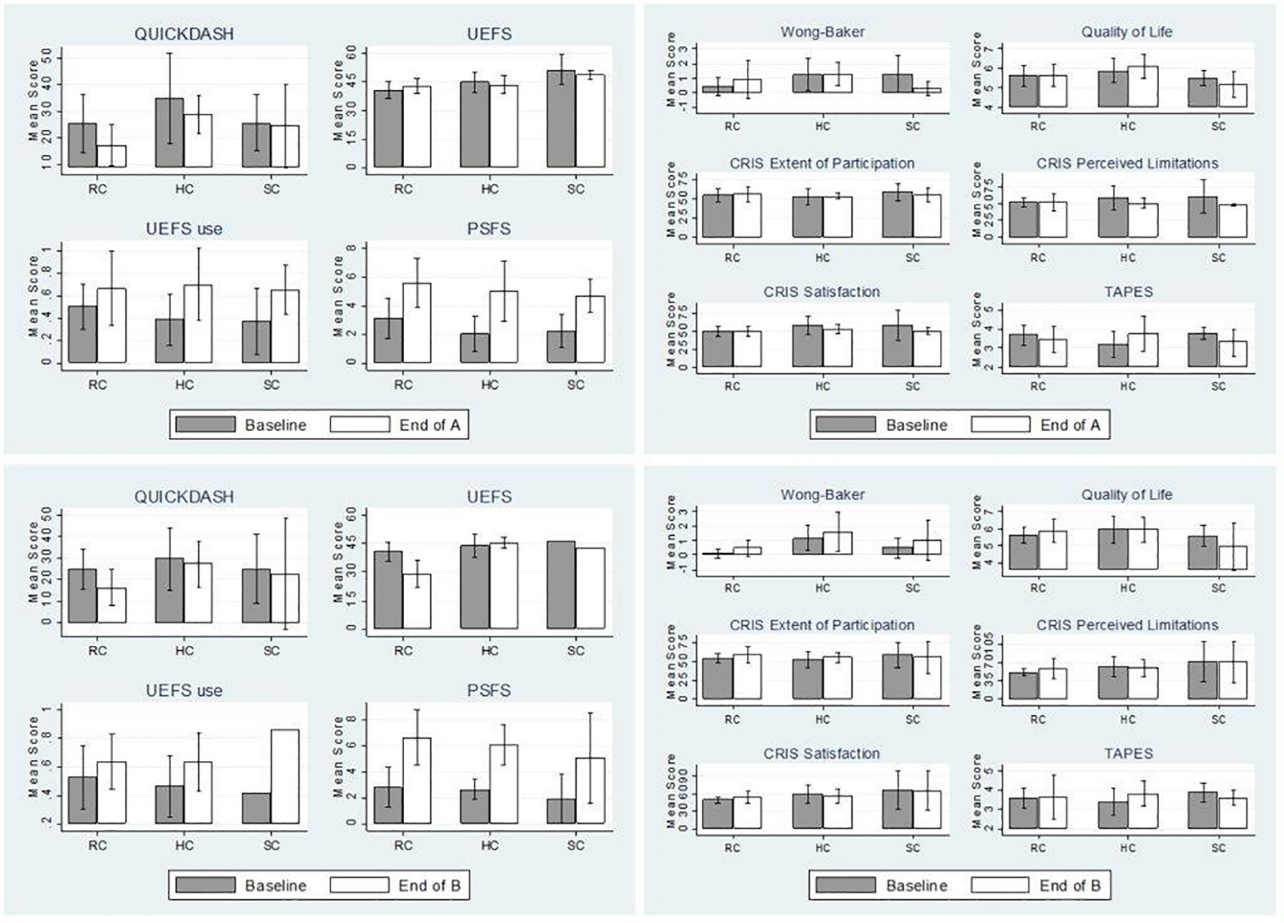
Self-report measures at Baseline compared to End of A and End of B by configuration level.

Fig 2A and 2B shows differences in self-report outcomes from Baseline to End of A and B by DEKA configuration level. QuickDASH scores declined (indicating less disability) for RC and HC users at the End of A, and for all 3 levels at the End of B. UEFS scores were very similar at Baseline and End of A, but clearly decreased (indicating less difficulty) for RC users at End of B. UEFS use scores improved for all configuration levels from Baseline to End of A and End of B. Finally, improvements of PSFS were consistent across configuration levels at both End of A and B.

Fig 2C and 2D show pain, quality of life, community integration and prosthesis satisfaction measures at each time point. Pain ratings increased for RC users and decreased for SC users at the End of A, but were elevated for all configuration levels at the End of B (differences not statistically significant after Bonferroni adjustment as mentioned above). Quality of Life improved slightly for HC users at End of A and for RC users at End of B, but was decreased for SC users. CRIS subscales were comparable or slightly from Baseline to End of A. However, from Baseline to End of B there was a small improvement in: RC users for all 3 subscales and in Extent of Participation for HC users. TAPES satisfaction scores indicated greater satisfaction with the prosthesis for HC users, but worse satisfaction for RC and SC users at the End of A. At the End of B, satisfaction of RC users was comparable to Baseline, satisfaction of HC users was higher than Baseline and satisfaction of SC users was slightly lower than Baseline.

## Discussion

This study built on prior research by comparing perceived function and functional performance of users of both conventional prosthesis and Gen 3 DEKA Arm. Whereas prior studies involved comparisons between the DEKA Arm and conventional prostheses after in-laboratory training, our analyses also compared outcomes after several months of home use. In addition, we compared other important outcomes that have not previously been examined, including community integration, pain, quality of life and several new activity measures.

After in-laboratory training, activity performance of the DEKA Arm and conventional prosthesis was equivalent, however after home use AM-ULA scores using the DEKA Arm surpassed conventional prosthesis scores, suggesting that home use after completion of formal prosthetic training may lead to better function. Given an ES of 0.71, we can interpret the effect of the DEKA Arm on improvement AM-ULA scores was moderate. We also found that activity performance with DEKA Arm, as measured by the T-MAP was moderately slower than performance with conventional prostheses at the end of in-laboratory training (ES = 07.6) but was not significantly slower after home use. These findings are consistent with our recent analyses of all home study subjects (including users and non-users of a conventional prosthesis) that showed improvement in AM-ULA scores with several months of home use. [36]

We did not observe differences in dexterity measures between DEKA and conventional prosthesis. An earlier study found that some measures of dexterity using the DEKA Arm were worse than using when using conventional prostheses.[5] However, that study included subjects who were using an earlier prototype of the DEKA Arm, the Gen 2, as well as the prototype used in the current study, the a Gen 3, The changes to hand and finger shape and foot controls [1] in the Gen 3 may, in part, explain differences in findings. A study comparing usability and satisfaction of DEKA found that in overall satisfaction, the Gen 3 was rated more favorably than the Gen 2, as was satisfaction with switching grips and the usability of all 6 grips. [37]

The DEKA Arm’s impact on perceived difficulty in activity performance and disability, as measured by the PSFS and QuickDASH, was evident after in-laboratory training and after home use experience. The improvements in the PSFS were large (ES 1.67 and 2.13 for Parts A and B respectively), while the improvements in the QuickDASH were moderate (ES 0.55 and 0.49 for Parts A and B respectively). Participants reported using the DEKA Arm to perform more activities (UEFS use) as compared to their conventional prostheses. The impact of the DEKA Arm on the UEFS was large (ES 1.18 and 1.0 for Parts A and B respectively). We did not observe differences between the DEKA Arm and conventional prostheses in the full group in measures of dexterity, prosthetic skill, spontaneity, pain, community integration or quality of life.

Improved PSFS and UEFS use scores are consistent, in part, with our earlier study which found that users of the DEKA Arm reported less difficulty in activity performance (PSFS) and engagement of the DEKA Arm in a greater proportion of daily activities (UEFS use).[5] Previous studies reported that the majority of DEKA users listed new activities that they could perform using DEKA that they were unable to do with their existing prosthesis.[38, 39] [40] Further, 65% of DEKA users preferred using DEKA Arm for tasks that they could also perform with their existing prosthesis.

An earlier study reported more spontaneity of prosthesis use (UNB test) with the DEKA Arm as compared to conventional prosthesis, however we did not observe this relationship in the current study.[5] We did find that comparisons between the DEKA Arm and conventional prosthesis at Baseline and End of A and B varied by configuration level. Unfortunately, our sample size of SC users who completed Part B testing was very small and data were missing for some tests, making comparisons of scores after home use challenging for this level.

Comparisons stratified by device type revealed similar patterns to findings for the overall group, though our analyses for the smaller sub-groups (body-powered users and single DOF users) were underpowered. Nevertheless, some important trends in the data were observed. Thus, our results should be interpreted as preliminary, and could be useful to other research groups planning studies that compare outcomes across device types. Our post-hoc tests estimating the sample size needed to achieve 80% power at an alpha of .05 showed dramatically different sample sizes needed for each outcome. Our comparisons for body powered users were adequately powered to compare T-MAP scores at End of A, but not at the End of B. At the End of B, comparisons of the AM-ULA outcomes were adequately powered for body-powered users. A larger sample would be required to be adequately powered for other outcomes (e.g. at the End of A JTHFT page turning requires an N of 9, QuickDASH requires an N of 16, etc.). Our comparisons between the DEKA Arm and myoelectric device required far larger samples at both End of A and End of B (End of A sample estimates range from N = 20 to N = 700). Similar findings were observed in post-hoc calculation of power for comparisons between single and multi-DOF devices and the DEKA Arm. The Baseline to End of B comparisons of single DOF users were adequately powered for the JTHFT feeding and AM-ULA tests, but other comparisons were under-powered. Our study had several other limitations. Our sample size, while relatively large for a study of a new upper limb prosthesis, was still very small, limiting statistical comparisons. Finally, our analysis of outcomes by configuration level was performed graphically, and so needs to be interpreted cautiously.

Although the majority of participants had improvements in function attributable to gaining home use experience, in analyses shown in another manuscript, functional gains appeared to plateau after month 2. [41] However, we cannot be certain that all participants had fully acclimated to using the device and that greater gains in function would not have been achievable with more experience. Thus, it is possible that our analyses have underestimated the impact of the DEKA Arm. Future studies with longer periods of home use would be needed to examine longer term outcomes.

## Conclusions

Participants using the DEKA Arm had less perceived disability and more engagement of the prosthesis in everyday tasks at the End of Part A, although their activity performance was slower. After home use experience, perceived disability was lower, prosthesis engagement higher, activity performance was improved and activity speed equivalent to using conventional prostheses. There were no differences between the DEKA Arm and conventional prostheses in measures of dexterity, prosthetic skill, spontaneity, community integration or quality of life.

While underpowered, comparisons stratified by device type and terminal device type revealed similar patterns to findings for the overall group. Comparisons between the DEKA Arm and conventional prosthesis by configuration level showed some variation by configuration level, but were limited by small sample sizes. The trends identified will be useful to other research groups planning studies to compare outcomes by device type.
